# Evaluation of the Effectiveness of Disinfectants on Impression Materials

**DOI:** 10.7759/cureus.54846

**Published:** 2024-02-24

**Authors:** Rajmohan Sivamani Chidambaram, Sudha Rajmohan, Pusulury Olive Prasad, Dorothy Kalyani, Rachappa Mallikarjuna, Shivakumar Ganiga Channaiah

**Affiliations:** 1 Department of Prosthodontics, Oman Dental College, Muscat, OMN; 2 Department of Preclinical Conservative Dentistry, Oman Dental College, Muscat, OMN; 3 Department of Orthodontics, Maratha Mandal Dental College, Belgaum, IND; 4 Department of Microbiology, Faculty of Dentistry, Meenakshi Academy of Higher Education and Research, Chennai, IND; 5 Department of Pedodontics and Preventive Dentistry, Oman Dental College, Muscat, OMN; 6 Department of Oral Medicine and Radiology, People's College of Dental Sciences & Research Centre, Bhopal, IND

**Keywords:** infection control, addition silicone, alginate, iodophor, sodium hypochlorite, disinfection, dental impression materials

## Abstract

Background

The disinfection of dental impression materials is a cornerstone of infection control in dental practice. This study aimed to evaluate the effectiveness of two disinfectants, sodium hypochlorite and iodophor, on alginate and silicone impression materials, which are prone to microbial contamination.

Methods

The study was structured into two main groups based on the impression material: Group I (alginate) and Group II (addition silicone), each further subdivided into two subgroups for disinfection with sodium hypochlorite and iodophor. For each subgroup, initial microbial swabs were taken before any treatment, followed by a second swab after rinsing and a final swab after disinfection. The mean colony-forming unit (CFU) counts, standard deviations, and standard errors of the mean were calculated for each stage of treatment.

Results

Prior to disinfection, Group I had a mean CFU count of 2,529.40, while Group II had a lower mean CFU of 1,417.40. After rinsing, there was a significant decrease in CFUs in both groups, with Group I at 1,337.10 and Group II at 415.10. Post-disinfection, Group I showed a mean CFU count of 73.00 for sodium hypochlorite and 0.00 for iodophor. Similarly, Group II achieved a CFU reduction of 99.00 with sodium hypochlorite and 0.00 with iodophor, demonstrating a marked reduction in microbial presence.

Conclusion

Iodophor was exceptionally effective in disinfecting both alginate and silicone impression materials, eliminating all detectable CFUs. Sodium hypochlorite also significantly reduced microbial counts but was not as effective as iodophor. Rinsing prior to disinfection was instrumental in reducing the microbial load, underscoring its importance in the disinfection protocol.

## Introduction

Contamination of dental impressions with varying amounts of blood and saliva is a routine occurrence in the dental office. Therefore, these impressions must be considered fomites with the potential to transmit serious diseases to all dental personnel who routinely handle them. It is therefore imperative that the recommendations for disinfecting dental impressions presented by the CDC [1] and the American Dental Association (ADA) [2] be followed for all patients.

According to the CDC’s information, chemical disinfectants like chlorine compounds, formaldehydes, glutaraldehydes, phenols, and iodophors have the potential to eradicate hepatitis B, herpes, and HIV in 10-30 minutes. Among these, glutaraldehydes in 2% solutions are accepted as effective disinfectants, but they should be in contact for no less than 20 hours to achieve an acceptable level of sterilization. Other acceptable disinfectants are sodium hypochlorite and iodophor [2].

In reviewing the literature to determine which disinfectant is recommended for the routine disinfection of all dental impressions, it was found that different impression materials need different disinfectants. Various studies found that the most commonly used disinfectant is sodium hypochlorite, although iodophor is the most suitable disinfectant for most of the impression materials. Therefore, this study was aimed at comparing the efficacy of the most commonly used disinfectant, sodium hypochlorite, with the most recommended disinfectant, iodophor. We also compared the disinfecting efficiency of iodophor and sodium hypochlorite on irreversible hydrocolloid impressions and analyzed the disinfecting prowess of the same agents, iodophor and sodium hypochlorite, on silicone impressions.

## Materials and methods

This study was conducted at Meenakshi Ammal Dental College and Hospital, Chennai, India.

Making of inoculums

A 24-hour culture of the *Enterococcus faecalis* strain (ATCC 29212) was mixed with 0.9% sterilized saline, and the turbidity was set to 0.5% using McFarland’s standard. This was done to make an inoculum with 1.5 × 108 organisms.

Preparation of the typodont model and making of impressions

An acrylic typodont model was immersed in a covered inoculum and left undisturbed for 10 minutes. It was then handled in an aseptic manner to make impressions using the two different impression materials. The first material was an irreversible hydrocolloid, widely recognized as alginate, which was sold under the trade name Plastalgin. This particular alginate was designed to be utilized with a single-mix technique, indicating that it did not require separate stages for mixing different viscosities of the material. Septodont was the producer of Plastalgin. The second material was an addition of silicone, a type of elastomeric impression material known by the trade name Affinis. This product came in two viscosities: putty and light body. Despite these differing viscosities, the Affinis was also meant to be used with a single-mix technique, implying a simultaneous or coordinated process for mixing and applying the material.

The set impressions were removed with a snapping motion, and the typodont model was autoclaved and inoculated again for further impression taking. A total of 40 such impressions were made out of irreversible hydrocolloid (alginate) and silicone.

Collecting the bacterial culture

We used a clean cotton swab (swab 1) to get a bacterial culture from the impression surface of tooth no. FDI 16 to see how many live bacteria were transferred from the dirty typodont to the impression.

The impression was then rinsed for 15 seconds using 250 ml of sterile water, simulating the rinsing of the impression that is typically performed in the dental work area. Excess water was shaken off, and another swab sample (swab 2) was taken from the impression surface of tooth no. FDI 26 to check the amount of viable bacteria still present after rinsing.

Both impressions were treated with a 1:10 concentration of 0.5% sodium hypochlorite and a 1:213 concentration of iodophor; alginate was subjected to spraying and preserved inside a zip lock pouch for 10 minutes; and silicone was immersed in disinfectant for 10 minutes (Table 1). Disinfection was performed at room temperature. The excess disinfectant was shaken off, and another swab sample (swab 3) was taken from tooth no. FDI 17. All three swabs were then directly plated on brain heart infusion agar using the lawn culture technique, and all plates were incubated at 37 °C for 18 hours (overnight).

**Table 1:** Disinfectant solutions used in this study

Product	Concentration	Methods of disinfection		Contact time (in minutes)	Manufacturer
		For alginate	For addition silicone		
Sodium hypochlorite	1:10 of 0.5%	Spraying	Immersion	10	Chen Chems, Chennai, India
Iodophor	1:213	Spraying	Immersion	10	Chen Chems, Chennai, India

Out of the total 20 alginate impressions made (Group I), 10 alginate impressions were disinfected with sodium hypochlorite (subgroup 1), and another 10 impressions were disinfected with iodophor (subgroup 2). A total of 30 swabs were made for each subgroup, and all the swabs were plated and incubated in the same manner.

Similarly, out of the total 20 addition silicone impressions made (Group II), 10 addition silicone impressions were disinfected with sodium hypochlorite (subgroup 1), and another 10 impressions were disinfected with iodophor (subgroup 2). A total of 30 swabs were made for each subgroup, and all the swabs were plated and incubated in the same manner (Table 2).

**Table 2:** Distribution of specimens

Group	Subgroup		Description
Group I: Alginate impressions	Subgroup 1: Disinfected with sodium hypochlorite	1a	Initial swab taken from infected alginate impression
		1b	Second swab taken from alginate impression after rinsing
		1c	Final swab taken from alginate impression after sodium hypochlorite disinfection
Group I: Alginate impressions	Subgroup 2: Disinfected with iodophor	2a	Initial swab taken from infected alginate impression
		2b	Second swab taken from alginate impression after rinsing
		2c	Final swab taken from alginate impression after iodophor disinfection
Group II: Addition silicone impressions	Subgroup 1: Disinfected with sodium hypochlorite	1a	Initial swab taken from infected addition silicone impression
		1b	Second swab taken from addition silicone impression after rinsing
		1c	Final swab taken from addition silicone impression after sodium hypochlorite disinfection
Group II: Addition silicone impressions	Subgroup 2: Disinfected with iodophor	2a	Initial swab taken from infected addition silicone impression
		2b	Second swab taken from addition silicone impression after rinsing
		2c	Final swab taken from addition silicone impression after iodophor disinfection

Measurement technique

The incubated plates were removed the next day after being incubated overnight, counted, and verified for the number of colonies formed using a digital colony counter (Toshiba Corporation, Tokyo, Japan). The colonies were confirmed under a microscope by their hemolytic properties and are observed as gram-positive cocci in pairs. The colony-forming units (CFUs)/ml calculation was performed as the number of colonies multiplied by 100.

Statistical analysis

SPSS software was used to analyze the readings. The mean, standard deviation, and test of significance were determined using IBM SPSS Statistics for Windows, Version 26.0 (Released 2019; IBM Corp., Armonk, NY, USA). A nonparametric Mann-Whitney U test was used to analyze the readings between the iodophor and sodium hypochlorite groups. A Wilcoxon signed rank test was used to analyze the readings for pre- and post-disinfection procedures. For all the evaluations, 0.005 was considered the level of significance.

## Results

The results are tabulated in Table 3. It was observed that there was a reduction in the number of CFUs following the rinsing and disinfection of the impressions. It is interesting to note that there is a complete elimination of CFUs after using iodophor disinfection.

**Table 3:** Mean pre- and post-disinfection CFUs for different impression material CFU, colony-forming unit

Group 1: Irreversible hydrocolloid						Group 2: Addition silicone					
Subgroup 1: Sodium hypochlorite			Subgroup 2: Iodophor			Subgroup 1: Sodium hypochlorite			Subgroup 2: Iodophor		
A	B	C	A	B	C	A	B	C	A	B	C
1,192	288	77	1,616	920	0	848	436	0	2,472	540	0
3,600	1,920	180	2,608	676	0	2,832	144	132	2,500	676	0
3,596	688	0	2,400	1,400	0	1,300	152	172	2,240	440	0
2,400	2,244	64	1,644	1,080	0	924	1,044	173	2,136	252	0
1,888	1,616	26	2,924	1,120	0	1,292	384	12	1,776	492	0
3,685	1,990	198	2,389	621	0	2,816	105	125	2,500	640	0
1,292	245	64	1,400	925	0	803	432	0	2,165	436	0
2,306	2,123	52	2,853	1,003	0	906	1,032	172	2,196	267	0
1,843	1,612	38	2,165	1,543	0	1,101	315	41	1,728	413	0
3,492	645	40	1,432	1,250	0	1,352	107	163	2,138	418	0

The CFU counts at different stages of disinfection were used to figure out how effective both irreversible hydrocolloid and added silicone were (Table 4).

**Table 4:** Observed effectiveness of the different disinfectants across the assessed compounds

Group	Subgroup	N	Mean	Standard deviation	Standard error of the mean
Sodium hypochlorite (before)	Irreversible hydrocolloid	10	2,529.40	989.659	312.958
Iodophor (before)		10	2,143.10	581.343	183.837
Sodium hypochlorite group (after rinsing only)		10	1,337.10	785.503	248.398
Iodophor group (after rinsing only)		10	1,053.80	292.471	92.488
Sodium hypochlorite (after disinfection)		10	73.00	62.695	19.826
Iodophor (after disinfection)		10	0.00	0.000	0.000
Before sodium hypo	Addition silicone	10	1,477.40	767.228	242.619
Before iodophor		10	2,185.10	271.847	85.966
After rinsing only sodium hypochlorite group		10	415.10	353.018	111.634
After rinsing only iodophor group		10	457.40	138.192	43.700
After disinfection with sodium hypochlorite		10	99.00	78.398	24.159
After disinfection with iodophor		10	0.00	0.000	0.000

The average number of CFUs in the irreversible hydrocolloid sample before it was cleaned with sodium hypochlorite was 2,529.40, and the standard deviation was a high 989.659, showing that the sample was very different. The standard error of the mean was 312.958, reflecting the precision of the sample mean estimate. In contrast, the iodophor group before disinfection had a lower mean CFU count of 2,143.10, a standard deviation of 581.343, and a standard error of 183.837, suggesting slightly less variability and more precision in the mean estimate. After rinsing only, the sodium hypochlorite group showed a marked reduction in the mean CFU count to 1,337.10, along with a substantial standard deviation of 785.503 and a standard error of 248.398. The iodophor group also exhibited a marked decrease in the mean CFU count to 1,053.80, with a notably smaller standard deviation of 292.471 and a standard error of 92.488, indicating a more consistent efficacy across samples.

Post-disinfection measures were most striking. The sodium hypochlorite group had a drastically reduced mean CFU of 73.00 with a much lower standard deviation of 62.695 and a standard error of 19.826, signifying a significant disinfection effect. The iodophor group after disinfection reached a mean CFU of 0.00 with no variability, as indicated by a standard deviation and standard error of 0.000, suggesting complete eradication of CFUs. For the addition of silicone samples, the mean CFU counts before disinfection with sodium hypochlorite were also high at 1,477.40, with a standard deviation of 767.228 and a standard error of 242.619. The iodophor group before disinfection had a higher mean CFU count of 2,185.10 but a lower standard deviation of 271.847 and a standard error of 85.966, pointing to more consistent results across this set of samples.

Following only rinsing, the sodium hypochlorite group for addition silicone showed a reduced mean CFU count of 415.10, with a standard deviation of 353.018 and a standard error of 111.634. Similarly, the iodophor group also displayed a decrease in mean CFU count to 457.40, along with a standard deviation of 138.192 and a standard error of 43.700. After the disinfection process, the addition of silicone samples treated with sodium hypochlorite had a mean CFU count of 99.00, a standard deviation of 78.398, and a standard error of 24.159. The iodophor-treated samples once again showed a mean CFU of 0.00 with no variability, as both the standard deviation and standard error were 0.000, indicating an effective disinfection outcome.

Table 5 shows the statistical analysis of the effectiveness of disinfectants on irreversible hydrocolloid and addition silicone conducted using the Mann-Whitney U test, Wilcoxon W test, and Z scores, with both asymptotic and exact significance (two tailed) assessed.

**Table 5:** Statistical analysis of the effectiveness of the different disinfectants across the assessed compounds

Statistical test	Material	Before	After rinsing	After disinfection
Mann-Whitney U	Irreversible hydrocolloid	40.500	37.000	5.000
Wilcoxon W		95.500	92.000	60.000
Z		-718	-983	-3.725
Asymptotic significance (two tailed)		0.473	0.326	0.000
Exact significance (two tailed)		0.481	0.353	0.000
Mann-Whitney U	Addition silicone	20.000	32.500	10.000
Wilcoxon W		75.000	87.500	65.000
Z		-2.269	-1.323	-3.414
Asymptotic significance (two tailed)		0.023	0.186	0.001
Exact significance (two tailed)		0.023	0.190	0.002

For the irreversible hydrocolloid, the Mann-Whitney U test values decreased from 40.500 before disinfection to 5.000 after disinfection, indicating a significant change. The Wilcoxon W test also showed a decrease from 95.500 to 60.000 in the same conditions. The Z scores, which were negative across all stages, became more pronounced after disinfection (-3.725), suggesting a strong disinfection effect. The asymptotic significance was not notable before and after rinsing only (0.473 and 0.326, respectively), but it was significant after disinfection (0.000). The exact significance mirrored this pattern, with values of 0.481 before and 0.353 after rinsing, but a definitive 0.000 after disinfection, confirming the efficacy of the disinfectant.

For the addition of silicone samples, the Mann-Whitney U test values ranged from 20.000 before disinfection to 10.000 after disinfection, indicating effectiveness. The Wilcoxon W test values also reflected a significant change from 75.000 to 65.000. The Z scores showed a significant negative value after disinfection (-3.414), suggesting effective disinfection. The asymptotic significance showed a significant result before disinfection (0.023) and after disinfection (0.001) but was not significant immediately after the rinsing only (0.186). The exact significance values were similar, at 0.023 before, 0.190 after rinsing only, and 0.002 after disinfection, corroborating the effectiveness of the disinfection process on addition silicone.

**Figure 1:**
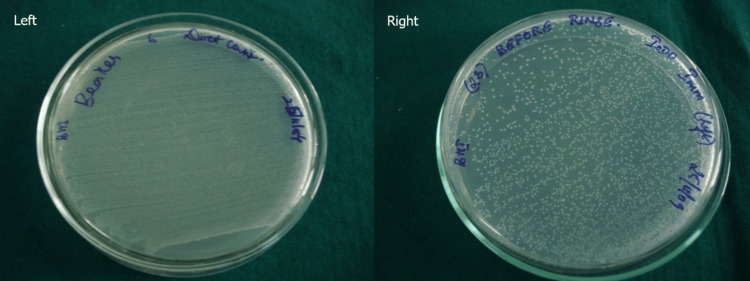
E. faecalis culture from the inoculum (left) and before rinsing (right)

Figure 1 represents the *E. faecalis* culture from the inoculum (left) and before rinsing (right).

**Figure 2:**
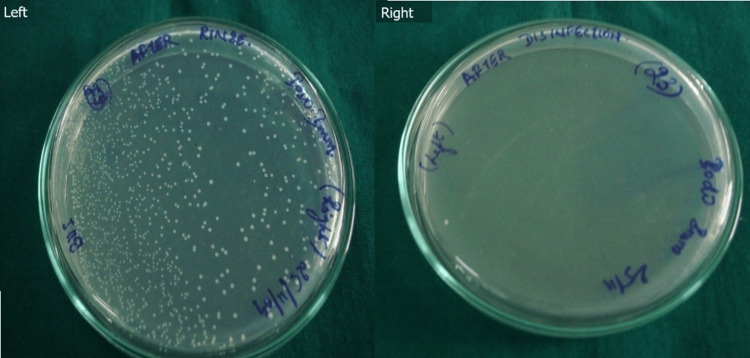
E. faecalis culture after rinsing only (left) and disinfecting with iodophor (right)

Figure 2, on the other hand, depicts the *E. faecalis* culture after rinsing (left) and disinfecting with iodophor (right).

## Discussion

*E. faecalis* was selected as a test organism because it is one of the most common oral bacteria that has inherent antimicrobial resistance, the ability to adapt to harsh environmental changes, and the ability to grow in biofilms [3-5]. Although the test would be more meaningful on HIV or Mycobacterium tuberculosis, this was not done due to its virulence properties; nonetheless, *E. faecalis* helped in deciding the effectiveness of both disinfectants on impressions.

In 1991, the ADA Council on Dental Materials, Instruments, and Equipment recommended immersion disinfection of irreversible hydrocolloid impressions in either “hypochlorite, iodophor, or glutaraldehyde with phenolic buffer” [6]. Several studies have been conducted on various disinfectants, especially on the method of usage, time of contact, effect of different disinfectants on different impression materials, and the resultant gypsum casts [7-9].

Of the recommended disinfectants, full-strength (5.25%) sodium hypochlorite was the most effective disinfectant with the shortest contact time (one minute) [10]. Chlorine-based disinfectants are reported to be the most effective of the ADA-recommended disinfectants [11]. When diluted 1:10 (0.525%), sodium hypochlorite is reported to have few, if any, negative effects on the quality of poured gypsum casts [12]. Therefore, a sodium hypochlorite dilution of 0.525% (1:10) was chosen for this study.

The iodine disinfectant was selected because it is recommended for all the impression materials as an appropriate disinfectant. The iodophor solution was freshly diluted (1:213) with distilled water to yield 75 ppm of titratable iodine. There are only a few articles that have compared the efficacy of sodium hypochlorite (the most common) with the most recommended disinfectant, iodophor [13].

Although the recommended method of disinfection is immersion, various studies have shown that there is a significant dimensional change in the irreversible hydrocolloid material [14] when it is immersed. Therefore, the spraying method was chosen in the study to disinfect as it does not affect the properties of alginate impression material. The study showed that both disinfection materials were effective with the spraying technique, but the iodophor showed superior disinfection (100%) by showing a clear elimination of *E. faecalis* totally.

The addition of silicone impression material showed no adverse effects while immersing it in a disinfectant solution [15-17]. Again, both disinfectants showed very effective disinfection with this impression material. However, iodophor proved to be significantly better once again (100%) than sodium hypochlorite in eliminating *E. faecalis* totally.

In this study, iodophor was found to be a better disinfectant than sodium hypochlorite with both impression materials. This is due to the fact that when full-strength sodium hypochlorite (5.25) is diluted to 0.525% to reduce the adverse effects on physical properties, there is a corresponding loss of antimicrobial activity, whereas iodophor, on the other hand, at this given strength (1:213) can act as a better disinfectant without causing any adverse effects on physical properties [18], even with the irreversible hydrocolloid. However, there are fewer studies that conclude that the use of iodophor produces casts of inferior quality [9], which may require further investigation.

Rinsing only before disinfection was noted to have significantly reduced the number of bacteria but not eliminated them completely. So, rinsing with running water was strongly recommended for all impressions before and after disinfection, before to have better disinfection and after to prevent remnants of disinfectant in the cast [19].

Further research is necessary to ascertain the fact that iodophor could be used as an effective disinfectant without affecting the quality of the gypsum casts. The quality of the impression materials has to be improved to meet the universal standards of disinfectants and not be left to the individual manufacturer’s choice.

## Conclusions

Our study concluded with several key findings regarding the efficacy of disinfectants on dental impression materials. Iodophor, when used at a 1:213 concentration, proved to be highly effective in eradicating colonies of *E. faecalis*, outperforming the 1:10 concentration of sodium hypochlorite. This was a significant result, as *E. faecalis* is known for its resilience and ability to survive in harsh environments, making it a challenging bacterium to eliminate. Further analysis revealed that the success of iodophor as a disinfectant was consistent, showing no significant variance in effectiveness when used on different types of impression materials. This uniformity in performance suggests that iodophor could be reliably used across various dental impression applications without concern for material-specific efficacy. Sodium hypochlorite, while recognized as an effective disinfectant for both alginate and silicone impression materials, did not surpass iodophor in terms of disinfecting power. Despite its acknowledged disinfection capabilities, sodium hypochlorite’s results were not superior to those achieved with iodophor. Additionally, the study underscored the importance of rinsing impressions before the disinfection process. This preliminary step was found to significantly reduce the number of microbial colonies present on the impressions, which, in turn, led to more successful disinfection outcomes. Based on these findings, the study recommended incorporating a rinsing step prior to disinfection as a best practice to enhance the overall effectiveness of the disinfecting procedure.

## References

[1] Mupparapu M, Kothari KR (2019). Review of surface disinfection protocols in dentistry: a 2019 update. Quintessence Int.

[2] Cottone JA, Molinari JA (1987). Selection for dental practice of chemical disinfectants and sterilizants for hepatitis and AIDS. Aust Dent J.

[3] Stuart CH, Schwartz SA, Beeson TJ, Owatz CB (2006). Enterococcus faecalis: its role in root canal treatment failure and current concepts in retreatment. J Endod.

[4] Love RM (2001). Enterococcus faecalis—a mechanism for its role in endodontic failure. Int Endod J.

[5] Rôças IN, Siqueira JF Jr, Santos KR (2004). Association of Enterococcus faecalis with different forms of periradicular diseases. J Endod.

[6] Almortadi N, Chadwick RG (2010). Disinfection of dental impressions - compliance to accepted standards. Br Dent J.

[7] Tan HK, Hooper PM, Buttar IA, Wolfaardt JF (1993). Effects of disinfecting irreversible hydrocolloid impressions on the resultant gypsum casts: part II—dimensional changes. J Prosthet Dent.

[8] Rentzia A, Coleman DC, O'Donnell MJ, Dowling AH, O'Sullivan M (2011). Disinfection procedures: their efficacy and effect on dimensional accuracy and surface quality of an irreversible hydrocolloid impression material. J Dent.

[9] Hamedi Rad F, Ghaffari T, Safavi SH (2010). In vitro evaluation of dimensional stability of alginate impressions after disinfection by spray and immersion methods. J Dent Res Dent Clin Dent Prospects.

[10] Qiu Y, Xu J, Xu Y, Shi Z, Wang Y, Zhang L, Fu B (2023). Disinfection efficacy of sodium hypochlorite and glutaraldehyde and their effects on the dimensional stability and surface properties of dental impressions: a systematic review. PeerJ.

[11] Hardan L, Bourgi R, Cuevas-Suárez CE (2022). Disinfection procedures and their effect on the microorganism colonization of dental impression materials: a systematic review and meta-analysis of in vitro studies. Bioengineering (Basel).

[12] Jasim ZM, Abass SM (2022). The effect of hypochlorous acid disinfectant on the reproduction of details and surface hardness of type III dental stone. Cureus.

[13] Sikka N, Brizuela M (2023). Glass ionomer cement. StatPearls [Internet].

[14] Rueggeberg FA, Beall FE, Kelly MT, Schuster GS (1992). Sodium hypochlorite disinfection of irreversible hydrocolloid impression material. J Prosthet Dent.

[15] Matyas J, Dao N, Caputo AA, Lucatorto FM (1990). Effects of disinfectants on dimensional accuracy of impression materials. J Prosthet Dent.

[16] Kotsiomiti E, Tzialla A, Hatjivasiliou K (2008). Accuracy and stability of impression materials subjected to chemical disinfection: a literature review. J Oral Rehabil.

[17] Memarian M, Fazeli MR, Jamalifar H, Azimnejad A Disinfection efficiency of irreversible hydrocolloid impressions using different concentrations of sodium hypochlorite: a pilot study. J Contemp Dent Pract.

[18] Tullner JB, Commette JA, Moon PC (1988). Linear dimensional changes in dental impressions after immersion in disinfectant solutions. J Prosthet Dent.

[19] Nassar U, Aziz T, Flores-Mir C (2011). Dimensional stability of irreversible hydrocolloid impression materials as a function of pouring time: a systematic review. J Prosthet Dent.

